# Cancer stem cells, epithelial-mesenchymal transition, ATP and their roles in drug resistance in cancer

**DOI:** 10.20517/cdr.2021.32

**Published:** 2021-06-17

**Authors:** Haiyun Zhang, Alexander Steed, Milo Co, Xiaozhuo Chen

**Affiliations:** ^1^Department of Biological Science, Ohio University, Athens, OH 45701, USA.; ^2^Edison Biotechnology Institute, Ohio University, Athens, OH 45701, USA.; ^3^Interdisciplinary Graduate Program in Molecular and Cellular Biology, Ohio University, Athens, OH 45701, USA.; ^4^Heritage College of Osteopathic Medicine, Ohio University, Athens, OH 45701, USA.; ^5^Department of Biomedical Sciences, Ohio University, Athens, OH 45701, USA.

**Keywords:** Tumor microenvironment, macropinocytosis, ATP internalization, ABC transporters, biological markers, apoptosis

## Abstract

The cancer stem cell (CSC) state and epithelial-mesenchymal transition (EMT) activation are tightly interconnected. Cancer cells that acquire the EMT/CSC phenotype are equipped with adaptive metabolic changes to maintain low reactive oxygen species levels and stemness, enhanced drug transporters, anti-apoptotic machinery and DNA repair system. Factors present in the tumor microenvironment such as hypoxia and the communication with non-cancer stromal cells also promote cancer cells to enter the EMT/CSC state and display related resistance. ATP, particularly the high levels of intratumoral extracellular ATP functioning through both signaling pathways and ATP internalization, induces and regulates EMT and CSC. The three of them work together to enhance drug resistance. New findings in each of these factors will help us explore deeper into mechanisms of drug resistance and suggest new resistance-associated markers and therapeutic targets.

## INTRODUCTION

Drug resistance in cancer is a very complicated process with numerous participating components. These include cancer cells in the tumor as well as stromal cells in the tumor microenvironment (TME) and their communications and interactions. Both cancer cells and TME are evolving and ever changing, making drug resistance a dynamic process. Within TME and cancer cells, proteins and small molecules are also involved in drug resistance, working as functional factors to regulate drug resistance. Our recent studies have shown that extracellular ATP (eATP) in TME is found in concentrations 10^3^ to 10^4^ times higher than eATP found in normal tissues^[1-5]^. The greatly elevated concentration of eATP plays much larger and diverse roles than previously realized in augmenting drug resistance in cancer cells through internalization^[6-8]^, promoting cell survival^[6-9]^, accelerating ATP-binding cassette (ABC) transporter activities^[10,11]^, inducing epithelial-mesenchymal transition (EMT)^[12]^ and possibly the formation of cancer stem cells (CSC). With these new advancements in mind, in this review, we summarize recent findings in these fields in an attempt to generate a clearer and more cohesive picture for CSC, EMT, ATP, and their distinctive and interactive relationships for creating increased drug resistance as a phenotype for cancer cells. Better anticancer therapies are likely to be developed by improved understanding of these factors and their roles in drug resistance.

## CANCER STEM CELLS

CSCs refer to a subset of tumor cells that have an unlimited self-renewal capacity and an intense tumorigenic potential (stemness) to differentiate into their non-tumorigenic progenies which comprise the rest of the tumor bulk^[13,14]^. Among tumor cells, CSCs are a subpopulation that constitutes varied percentages of the total tumor cells, and this fraction can dramatically increase upon anticancer therapy correlating with therapy resistance and tumor rebound^[13,15]^. CSCs are believed to reside stochastically in a tumor but be predominantly induced in hypoxic, low pH, and fewer nutrient regions such as the tumor niche^[16]^. One study discovered that cancer cells can reprogram into CSCs when receiving DNA damage signals, suggesting the potential risk of genotoxic therapies in inducing CSC and tumor relapse^[17]^. Substantial studies have shown that CSCs have enhanced resistance to conventional radio-/chemo-therapy and targeted therapies and play essential roles in cancer relapse and metastasis^[14,18]^. Tumors with a high CSC signature tend to have a poorer prognosis compared with those with decreased CSC populations^[19]^. CSCs have the nature to be slow-cycling or quiescent, spending the majority of time in G0 and thus can circumvent the chemotherapy or radiotherapy targeting the rapidly dividing tumor cells^[20]^. Other cellular mechanisms of therapy resistance in CSCs will be discussed in the following sections.

The intratumoral heterogeneity and its implication in therapy resistance (tumor recurrence) was explained by two models: clonal evolution (CE or stochastic) and CSC (hierarchy). In the CE model, tumor cells all have equivalent potential to accumulate mutations over time to gain resistance-related traits. The selection of the traits leads to increased tumor heterogeneity and disease progression^[21]^. On the contrary, in the CSC model, tumors are initiated with a heterogeneous mixture of genetically distinct subclones of CSCs that each gives rise to their fast-dividing progeny cancer cells and ultimately leads to the functional and phenotypical heterogeneity of the tumor^[22]^. According to the CSC model, CSCs contribute to drug resistance and tumor progression by the intrinsic resistance possessed by CSCs combined with genetic changes that occur during therapy^[22]^. The main target of conventional cytotoxic therapies are the rapidly-dividing and apoptosis-sensitive cancer cells, but resistant CSCs can survive, enrich in number, accumulate oncogenic genetic changes during therapy and finally expand even under drug treatment, functioning as the precursors of new resistant tumor masses and ultimately leading to clinical relapse^[22-24]^. More recently, several studies questioned the unidirectional hierarchic CSC model by showing cancer cells are plastic^[25]^. A famous example is that cell population isolated from breast cancer cell lines displayed stem-, basal-, or luminal-like phenotypes^[26]^. Each phenotype was capable to convert into the other two phenotypes and over time produced all three phenotypes with the same proportions of cell types in the original cell line, but only stem-like cells could form tumors upon xenotransplantation^[26]^. One recent study showed that glioblastoma cells expressing CSC markers represented a plastic state that can be adopted by most of the cells in response to various microenvironmental cues, which may contribute to enhanced tumorigenic potential^[27]^. With the CSC plasticity, a more fluid hierarchic CSC model is now accepted, where cancer cells can undergo a dynamic transition from a non-CSC to CSC phenotype and vice versa to adapt to different stimuli in TME^[25,28]^. The activation of EMT serves as one of the mechanisms for switching phenotypes.

### ABC transporters are functional markers of CSCs

Forty-nine ABC transporters have been identified in the human genome and are classified in seven subfamilies (*ABC A-G*) based on amino acid sequence similarities and protein structural organization^[29]^. Most ABC transporters are membrane-bound and function as active transporters that catalyze the transport of diverse substrates, including anticancer agents across the plasma and intracellular membrane, using ATP-provided energy. Three major ABC transporters are frequently implicated in multidrug resistance (MDR), namely, P-glycoprotein (P-gp/*ABCB1*), breast cancer resistance protein (BCRP/*ABCG2*), and multidrug resistance-associated proteins (MRPs/*ABCC*s). These ABC transporters can mediate resistance to a wide range of chemotherapeutics, EGF receptor-tyrosine kinase inhibitors (EGFR-TKIs) and other targeted drugs^[30]^, thereby predicting recurrence and poor survival^[31,32]^. Moreover, co-expression of multiple ABC transporters correlates with decreased overall survival^[33]^. The robust cell-surface expression of ABC transporters, especially P-gp and BCRP, is recognized as a key feature of CSCs^[13]^. Patrawala *et al*.^[34]^ first characterized “side population” cells from human cancer cells purified by flow cytometry-based technique isolating for the side population of cells which exclude dye via ABC transporters. These side population cells possessed stem cell properties mediated by BCRP^[34]^. As of now, several studies have demonstrated P-gp and BCRP play a key role in maintaining cell viability as well as stemness features^[35-38]^. On the other hand, the expression of ABC transporters is under the regulation of multiple signaling pathways associated with CSC phenotypes and EMT activation, contributing to CSC and EMT-related resistance. This will be described in later sections. Although potentially life-threatening adverse events remain a major obstacle for the first-, second- and third-generation of small molecule ABC transporter inhibitors, therapies combating ABC transporter-mediated drug efflux continue to be developed^[39]^. One advanced strategy is to use TKIs as ABC transporter inhibitors, because most TKIs (e.g., imatinib, erlotinib, nilotinib and lapatinib) can target more than one ABC transporter and such a multi-target approach might be promising^[39]^. Another strategy is to deplete intracellular ATP molecules, which cuts down the energy supply of ABC transporters and reduces MDR. Several approaches of this strategy will be discussed in section V.

### ALDH-associated drug resistance

The ALDH family of proteins are cytosolic enzymes that scavenge intracellular highly toxic aldehydes to carboxylic acids. ALDH activity measured by Aldefluor assay is a hallmark of CSCs. Though these proteins have minimal metabolic activity in normal cancer cells, quiescent CSCs exhibit high ALDH activity to catalyze drug metabolism and turn the drugs into less toxic agents^[13]^. Among 19 members of ALDHs, aldehyde dehydrogenase 1 (ALDH1) is considered a CSC biomarker, which indicates stemness and resistance to chemotherapy^[40,41]^ and predicts poor clinical outcomes in breast and prostate cancer^[42]^. High expression of ALDH in CSCs have been characterized to play key roles in resistance to chemotherapeutics (cisplatin, doxorubicin, etoposide, and fluorouracil), TKIs (Gefitinib and Erlotinib) and radiation in various cancer cell lines including breast, lung, gastric, and head/neck cancers^[42]^. High ALDH activity also correlates with EMT, tumor invasion and metastasis. Ovarian cancer stem-like cells sorted by high expression of ALDH are prone to activate stemness, invasiveness, EMT and anti-apoptotic properties compared to those with low ALDH^[43]^. Mechanistically, ALDH1 and aldehyde dehydrogenase 3 family member A1 (ALDH3A1) can act as a detoxifying enzyme and protect cells from increased reactive oxygen species (ROS) by direct scavenging of radiation-induced free radicals or by producing the antioxidant reduced nicotinamide adenine dinucleotide phosphate (NADPH), suggesting a pivotal role in resistance to radiation and chemotherapy^[44]^. High ALDH1 family member A1 (ALDH1A1) led to nuclear factor-erythroid 2-related factor 2 (Nrf2) activation via p62-associated pathway in ALDH1-high CSC-like ovarian cancer cells. Nrf2 activation in these cells contributed to CSC-like properties including high levels of P-gp and BCRP and chemo resistance^[45]^. Additionally, enhanced ALDH activity is associated with the activation of the pro-survival signaling pathways such as transforming growth factor beta (TGF-β), platelet-derived growth factor (PDGF), Notch, and the mechanistic target of rapamycin (mTOR)^[42]^. For example, ALDH activity was regulated by another putative CSC marker Nanog, through the Notch1/Akt signaling pathway in breast cancer stem cells^[46]^. Additionally, the enhanced ALDH activity led to cellular radio-resistance by simulating double-strand break repair^[46]^. Inhibitors directly targeting isoforms of ALDH, such as ALDH1A1 and ALDH3A1 have been developed, which hold promise for eliminating CSCs particularly in gynecologic cancers^[47]^. Challenges of using ALDH inhibitors include limited *in vivo *activity and the need of using ALDH inhibitors in combination with other anticancer therapies^[47]^.

### Oxidative stress-associated resistance

ROS can induce therapy resistance at both high and low levels^[13,48]^. Compared to more differentiated cells, CSCs have a robust ROS scavenging system and maintain ROS at low levels, which may be attributable to an increase in ROS scavenging molecules or/and enhanced mitochondrial respiratory capacity^[49-51]^. On the one hand, the low ROS levels serve to prevent cell death from ROS overloading and maintain stemness, tumorigenic capacity and tumor radio- and chemo-resistance^[48,50-53]^. On the other hand, elevated ROS levels emerge as a potential factor to induce EMT particularly under hypoxia, and such induction of EMT can contribute to the acquisition of stemness features^[48,54]^. Whereas excessive oxidative stress can cause damage to DNA, proteins and lipids and lead to apoptosis^[55]^.

Glutathione (GSH) is vitally important for the maintenance of cellular redox homeostasis and is important for various signaling processes in cell apoptosis and proliferation^[56]^. Survival through radio- and chemo-therapy may trigger the synthesis of GSH at high amounts, which can enhance cell survival^[19]^ as well as function as a signaling molecule to induce CSC phenotype^[57]^, thus serving as one way to integrate ROS with CSC signaling^[57]^. Interestingly, one study reported that exogenous administration of GSH can increase intracellular GSH levels and induce resistance to cisplatin by suppressing apoptosis^[58]^. GSH conjugation is the first step of the cell detoxifying mercapturic acid pathway, in which Glutathione S-transferases (GSTs) binds to GSH and cytotoxic compounds (include certain chemotherapeutics), forming conjugates readily excreted via membrane transporters^[56]^. Several members of the MRP transporter family are responsible for this excretion, including MRP1, MRP2, MRP3, MRP4, MRP5, MRP7^[56]^. Moreover, ABC transporters such as MRP1 are often upregulated in drug-resistant cancer cells along with GSH or GSTs^[59,60]^, whereas some ABC transporters like *ABCB6* can regulate ROS via porphyrin biosynthesis and diminish the ROS-inductive effect of chemotherapeutics^[61]^. Notably, the overexpression of GSTs and MRP1 has been recognized as one underlying mechanism for therapeutic resistance in various cancers (breast, colorectal, lung, ovarian, pancreatic), with overexpressed GST pi 1, a member of the GST family, showing intense correlation with CSCs-related resistance and being considered as a biomarker for cancer^[56,62-65]^.

### Metabolic reprograming, CSC plasticity, and hypoxia

Cancer cells display a rapid metabolic adaptation in response to low-nutrient and hypoxia in the surrounding tumor microenvironment. This adaptation, namely “metabolic reprogramming”, refers to the shift of cellular bioenergetics, a new hallmark of cancer^[66]^ and is utilized by CSCs^[67]^. CSCs can fulfill their energy demands by shifting their phenotypes^[13]^. The CSC phenotype is believed to be a plastic state that can be adopted by most cancer cells^[27]^ and is capable of transitioning between a stem, mesenchymal-like phenotype and a non-stem, epithelial-like one^[68]^. Different CSC phenotypes can correlate with metabolic differences and add to the heterogeneity of cancer cells, and thereby ease their adaptation to TME and limit the efficacy of conventional therapies^[27,69]^. Activation of EMT in non-CSCs is a predominant driver to convert them into the CSC state^[70]^. EMT induces metabolic reprogramming to accommodate cellular changes during EMT, and imparts cancer cells with the phenotypic and metabolic plasticity of CSC, which is required for drug resistance.

Hypoxia has been recognized as a key factor in TME that contributes to the plasticity and heterogeneity of CSCs^[71]^. Cancer cells localized in hypoxic regions tend to display CSC-like phenotypes and are more invasive than cells in oxygenated regions within tumors^[72]^. The non-CSCs grow and proliferate rapidly, which leads to hypoxia in the nearby milieu^[13]^. The poor oxygen availability may reasonably shift the metabolism from disadvantaged oxidative pathways to glycolysis^[73]^.

Chemoresistant pancreatic cancer cells rely on glycolysis to maintain low ROS levels and consequent CSC and EMT phenotypes^[74]^. This correlation of hypoxia and CSC features have been demonstrated in various tumor types^[73]^. One central mediator of this adaptive metabolic shift is hypoxia-inducible factor 1 (Hif-1)^[75]^. Hif-1α is stabilized under hypoxia and can stimulate glycolysis by upregulating glycolytic enzymes such as GLUT1, lactate dehydrogenase A, and pyruvate dehydrogenase kinase 1 (PDK1)^[28]^. Further, Hif-1α promotes CSC formation and maintenance under hypoxia by activating Notch signaling in glioblastomas^[76]^. Hif-1α is also an emerging activator EMT under hypoxia^[77,78]^ and the activation of EMT triggers the conversion to CSCs^[27]^. Moreover, Hif-1 shifts metabolic flux to enhanced glycolysis, serine synthesis pathway and mitochondrial one-carbon metabolism to increase mitochondrial antioxidant production (NADPH and glutathione), serving to reduce mitochondrial ROS levels and thus coupling redox regulation with the induction of CSC phenotype^[75]^. Besides, Hif-1α is a crucial transcriptional activator of P-gp and has implications in enhanced activities of MRP1 and BCRP, contributing to chemoresistance^[79]^. Along with Hif-1, El-Sahli *et al*.^[67]^ summarized other pathways involved in metabolic reprogramming of CSCs. Among which, yes-associated protein 1/transcriptional coactivator with PDZ-binding motif (YAP/TAZ), Janus kinases/signal transducer and activator of transcription proteins (JAK/STATs), and nuclear factor-κB (NF-κB) are also implicated in drug resistance^[67,80]^.

Furthermore, the metabolic reprogramming of CSCs is a flexible mechanism which allows CSCs to either rely on glycolysis or mitochondrial OXPHOS, depending on the tumor type and the adaptation to a particular condition (such as hypoxia) within the TME^[13]^. The metabolic switch to glycolysis was demonstrated in CD44^+^CD24^low^EPCAM^+ ^CSCs in breast cancer, radioresistant CSCs in nasopharyngeal carcinoma, and CD133^+^CD49f^+ ^CSCs in hepatocellular carcinoma. The underlying mechanisms include Hif-1α-mediated enhanced glycolysis, STAT3-mediated induction of aerobic glycolysis^[81]^, and c-Myc-driven glycolytic programme^[82]^. In contrast, CSCs in other cancer types adopt OXPHOS as the preferred process for energy production. This notion is exemplified in CD133^+^ CSCs in glioblastoma and pancreatic adenocarcinoma, low reactive oxygen species quiescent stem cells in leukemia, and side population of lung and breast cancer^[83]^. One study demonstrated diverse oncogene-addicted cancer cells and CSCs (lung, melanoma, leukemia, breast) showed selective reliance on OXPHOS and resistance to EGFR-TKIs^[84]^. Invasive ovarian cancer and ovarian CSCs were also recently found to prefer OXPHOS, and chemoresistant ovarian cancer cells exhibited adaptability to switch between OXPHOS and glycolysis under different stress^[85]^. In addition to glycolytic and OXPHOS phenotypes, CSCs can also rely on mitochondrial fatty acid oxidation for ATP and NADPH generation^[83]^.

Given the crucial role of hypoxia in CSC metabolism and plasticity, targeting Hif-1α and downstream Hif signaling pathways to overcome hypoxia is a reasonable approach to reduce CSCs. Strategies inhibiting Hif-1α has been well summarized by Onnis *et al*.^[86]^. Inhibition of glycolysis also serves as a promising approach in combination with chemotherapy, which was shown to effectively eradicate chemoresistant glioblastoma CSCs which reside in their hypoxic niches and rely on glycolysis for ATP generation^[87]^. Additionally, inhibition of glycolysis by dichloroacetate may reverse the metabolic shift to OXPHOS, leading to increased ROS and promoted apoptosis in CSCs of several cancer types^[72]^.

### Acidic TME induces CSC phenotypes and chemoresistance

Acidosis, meaning extracellular pH ranging from 6.2 to 6.8, is a hallmark of TME in solid tumors^[88]^. Tumor acidosis is a consequence of the exacerbated aerobic glycolysis and hydration of CO_2_ derived from mitochondrial respiration^[89]^. Acidosis can activate EMT by modulating the expression of EMT-related proteins^[90]^. The adaptive CSC-like phenotypes are likely triggered and maintained by the EMT activation^[91,92]^. When exposed to acidic conditions, cancer cells have been reported to show reduced proliferation and even quiescence, and increased resistance to apoptosis^[93]^. Acidosis may regulate CSC-associated gene expression via hypoxia-inducible factor 2α (Hif-2α)^[91]^, which is considered as a master regulator of gene expression and a marker of CSC in glioma^[94]^. Both the abundance and transcription activity of Hif-2α was documented to be increased by acute acidosis^[91]^. Acidosis is related to the metabolic shift towards a dependence on mitochondrial OXPHOS, which is an adaptative mechanism for efficient ATP generation^[95]^, particularly in therapy-resistant CSCs of certain types of cancers as described above. Altered lipid metabolism is another result of acidosis and is correlated with chemoresistance. For instance, acidosis activated partial EMT and increased ATP generation through enhanced fatty acid uptake in various cancer types^[96]^. Additionally, under acidosis, increased lipid desaturation was found to be essential to the maintenance of stemness in ovarian cancer cells^[97]^. Acidosis can also contribute to resistance by enhancing drug efflux activities of P-gp^[98]^ and BCRP^[99]^. Finally, cancer cells undergo autophagy as a survival mechanism when stimulated by acute acidosis^[91]^, and this increased autophagic flux is associated with stemness, quiescence, and chemotherapy resistance in a variety of cancers^[100]^.

The adaptive metabolism under tumor acidosis sustains resistance-related CSC phenotypes, which can be targeted by inhibiting key enzymes/transporters in the metabolic pathway. Moreover, many pH sensitive nanoparticles have been developed with the goal to selectively deliver anticancer drugs to TME^[101]^. A combination of such a delivery system and siRNA of key enzymes/transporters (e.g., ASCT2) might significantly inhibit tumor growth and reduce drug resistance^[102]^.

### Autophagy in CSCs and resistance

Autophagy is an arising player in the maintenance of CSC stemness^[100]^. Autophagy can be stimulated by the same range of stressors in CSC and EMT induction, including oxidative stress, hypoxia, nutrient-deprivation, as well as genotoxic therapies^[48,100]^. Many EMT inducers like TGF-β and CSC related transcription factors such as sex determining region Y-box 2 (SOX2), and Nanog are also capable of inducing autophagy^[100]^. On the other hand, CSCs show dependence on autophagy. Autophagy promotes the expression of stem cell markers CD44 and mesenchymal markers vimentin, as well as *in vivo *tumorigenesis and CSC related drug resistance^[100]^. Autophagy acts as a double-edged sword for resistance to therapies: it protects cancer cells from stressors by recycling metabolites degraded from damaged organelles and mediates MDR, thus protecting cancer cells from anticancer drugs. On the contrary, it may also induce autophagic cell death and thus kill MDR cancer cells with deficient apoptosis pathways^[30,100]^.

Forkhead box (FOX) O3a, a transcription factor suppressing protective autophagy particularly in CSCs, was identified to form a negative feedback loop in which its turnover was controlled by autophagy. Upon autophagy inhibition, FOXO3a protein increased and accumulated in the nucleus, and transcriptionally activated the pro-apoptotic gene, p53 upregulated modulator of apoptosis, leading to p53-independent apoptosis and consequently sensitizing colorectal cancer (CRC) cells to chemotherapy drugs such as doxorubicin and etoposide^[103]^. This study suggested that a homeostasis of autophagy is maintained by FOXO3a and is crucial to cancer cell survival. In contradiction to what is described above, FOXO3a was reported to play a promoting role in both hypoxia-induced and sorafenib-induced autophagy and sorafenib resistance in hepatocellular carcinoma, while the knockout of FOXO3a significantly inhibited autophagy and restored sorafenib sensitivity^[104]^.

Nrf2 is an important activator of protective autophagy. In response to chemotherapy-induced oxidative stress, the accumulation of Nrf2 transcriptionally upregulates genes involved in the activation of protective autophagy such as an autophagy cargo receptor p62. Such autophagy eliminates cellular cytotoxic products and thus promotes tumor survival and chemoresistance^[105]^. High CD44 expression in breast cancer stem-like cells led to p62-associated Nrf2 elevation, possibly due to the activation of autophagy. Nrf2 activation consequently contributed to the promotion of tumor initiating capability, migration and invasion, and anticancer drug resistance^[106]^. In leukemia stem cells, inhibition of histone methyltransferase G9a induced ROS and apoptosis, which was bypassed by the activation of the protein kinase RNA-like endoplasmic reticulum kinase (PERK)/Nrf2 pathway^[107]^. Autophagy was also found to be induced upon G9a inhibition and autophagy inhibitors significantly increased apoptosis, suggesting PERK/Nrf2 might suppress ROS-induced apoptosis by induction of pro-survival autophagy, although the autophagy was not abrogated by PERK/Nrf2 inhibition^[107]^.

Mitophagy is one of the mitochondria quality control systems which selectively targets damaged mitochondria for autophagy-mediated degradation. Mitophagy can lead to reduced ROS and promote cell survival and resistance in cancer cells^[108,109]^. In CSCs, mitophagy is involved in the maintenance of stemness by suppressing mitochondria-mediated p53 phosphorylation and allows the expression of downstream CSC transcription factor Nanog^[110]^. Mitophagy also prompts a quiescent state by enhancing mitochondrial turnover and forces the energy metabolism from OXPHOS to glycolysis^[100]^. A recent study found that mitophagy can be induced by doxorubicin in CD133^+^/CD44^+^ CRC CSCs, and inhibition of mitophagy significantly enhanced doxorubicin sensitivity, suggesting a role of mitophagy in CSC-related chemoresistance^[111]^. Autophagy and mitophagy are believed to be maintained in equilibrium in CSCs to promote resistance, therefore either autophagy/mitophagy inhibition or overactivation in order to disrupt the equilibrium would be a therapeutic strategy^[13]^, Table 1 summarizes drug resistance factors discussed in this section.

**Table 1:** Major factors involved in cancer stem cell-related drug resistance

**Associated factor**	**Major normal biological function**	**Functions in cancer drug resistance**	**Major Ref.**	**Additional remarks**
**ABC transporters**	Efflux toxic metabolites	Efflux anticancer drugs, reducing ROS	[30,31,33,35,38,56,62,64] [61]	Functional marker of CSC
**Low oxidative stress**	Anti-apoptosis, detoxification	Maintaining stemness, preventing therapy-induced apoptosis	[48,50,51] [19,49,50,53,56,58]	
**ALDH**	Oxidation of aldehydes, detoxification	Detoxification, reducing ROS, inducing CSC	[13,41,42] [42,44] [45]	Hallmark of CSC
**Metabolic reprogramming**	Not applicable	Reducing ROS, adaptation to TME	[73,75] [13,27,69,84,85]	Hallmark for cancer metabolism
**Autophagy**	Removal and recycling cellular components	Inducing CSC, promoting survival	[48,100] [30,100,103,105-109]	Mitophagy reduces ROS and promotes survival

## EPITHELIAL-MESENCHYMAL TRANSITION

The mechanisms taken by CSCs to confer drug resistance are believed to be largely attributable to the activation of EMT^[70]^. EMT is a development process during embryogenesis and epithelial wound healing. As hijacked by cancer cells, EMT endows epithelial cells with malignant traits such as loss of cell-cell adhesion and apical-basal polarity, transition to a mesenchymal phenotype, and enhanced migratory and invasive potentials. EMT can be triggered by hypoxia^[112]^, mechanical stress^[113]^, and inflammatory cytokines (e.g., TGF‐β, TNF‐α, IL‐1β, IL‐6)^[114]^, among which TGF-β/SMAD signaling is established in multiple EMT models to play a central role^[70]^. Cells undergoing EMT display decreased expression levels of epithelial markers such as E-cadherin, zonula occludens-1 (ZO-1), occluding, laminin-1, claudin, and increased mesenchymal markers such as N-cadherin, vimentin and fibronectin^[115]^. Such a switch in gene expression is under the control of multiple key signaling pathways, including TGF-β, Notch, Wnt, Hedgehog (Hh), PI3K/Akt/mTOR, MAPK/ERK, NF-κB^[115]^. These signaling pathways ultimately converge on a relatively small group of transcription factors that orchestrate the changes in gene-expression associated with EMT^[70]^. These transcription factors are often referred to as “EMT-inducing transcription factors”, such as Snail, Slug, zinc finger E-box binding homeobox 1/2 (Zeb1/2), Twist1/2, FOXC2, and FOXM1. Besides transcriptional control, EMT is also regulated by post-transcriptional mechanisms, including microRNAs, long non-coding RNAs, alternative splicing, epigenetics, and post-translational protein stability^[116]^.

An important notion brought by Shibue and Weinberg^[70]^ is that the EMT program is usually partially activated and concurrently expresses epithelial and mesenchymal markers, and the partial EMT phenotypes are highly diverse even within individual tumors. Remarkably, cancer cells that undergo partial EMT are recognized to have a higher metastatic risk than complete EMT, probably due to cell migration by clusters and enhanced attachment to the extracellular matrix^[70,116]^. The induction of partial EMT phenotype is also associated with CSC expansion^[117]^. Jolly *et al.*^[116]^ summarized evidence for the existence of the partial EMT phenotype and its implication in resistance to chemo- and targeted therapies in multiple carcinomas.

### EMT induces CSC phenotypes and resistance

EMT is a critical regulator of CSC phenotypes, with emerging evidence indicating therapy-resistant cells enter the CSC state via the activation of EMT^[70]^ and thus display resistance to radio- and chemo-therapy by the gained CSC properties. Though the elucidation of the precise mechanisms of how EMT induces stemness needs further efforts, current studies suggest several EMT-TFs can promote stemness via the activation of CSC regulatory signaling pathways (Notch, Hh, Wnt, Mitofusin) and epigenetic regulators such as microRNA (miR)-200 family members and polycomb complex protein BMI1, a key player in CSC maintenance^[118]^. For instance, Slug enhances CSC self-renewal capacity; Zeb1 can directly inhibit miR-200 family members and induce stemness by Zeb1/miR-200/BMI1 or Zeb1/miR-200/Notch axis; Twist transcriptionally activates BMI1^[118]^. One newly recognized connective player is the mitochondrial fusion pathway: upregulation of mitofusin proteins can be triggered by EMT and lead to segregation of the fused mitochondria into CSC upon asymmetric cell division, which provided CSCs with enhanced glutathione synthesis and ROS scavenging to maintain stemness^[52]^. In turn, CSCs can provoke EMT in non-CSCs via exosome-mediated mechanism and shape the latter to CSCs with an apoptosis-resistant feature^[119]^.

The cellular mechanisms of resistance tightly linked to EMT are attributable to the roles of TGF-β and some EMT-TFs in activating pro-survival signaling, enhancing DNA damage repair, and promoting MDR by ABC transporters. TGF-β and EMT-TFs are also implicated in CSC induction, maintenance of stemness and modulation of metabolism, demonstrating the links among EMT, CSC, and drug resistance.

#### TGF-β associated drug resistance

TGF-β is a well-established EMT-inducing cytokine that functions as a double-edged sword in relation to drug resistance. At early stages of tumor progression, TGF-β functions as an important tumor suppressor in epithelial cells by inducing apoptosis; however, during later stages, the pro-metastatic role of TGF-β often contributes to resistance^[120,121]^. A recent study revealed one mechanism of opposing responses of cell survival and apoptosis mediated by TGF-β: by alternative splicing of TGF-β-activated kinase 1 (Tak1). The short isoform of Tak1 was constitutively active and supported TGF-β-induced EMT and NF-κB signaling, whereas the full-length isoform of Tak1 promoted TGF-β-induced apoptosis^[122]^. Chronic exposure to chemotherapy induced an increase in TGF-β, EMT markers, stem cell markers CD44^+^/CD24^-^, sphere formation, and anti-apoptosis protein Bcl-2 in triple negative breast cancer cells, indicating a role of TGF-β in chemo-resistance by regulating EMT, stemness, and apoptosis^[123]^. TGF-β prompts EMT in epithelial non-CSCs, and the activated EMT-regulatory genes can be crucial in driving cellular plasticity towards mesenchymal CSC states. This is exemplified by Zeb1: in non-CSCs the transcription of Zeb1 was maintained in a poised chromatin configuration which readily enabled the conversion to a stem-like state in response to TGF-β^[124]^. Moreover, TGF-β can modulate CSC metabolism and promote tumor heterogeneity, leading to drug resistance^[125]^. For instance, a high concentration of TGF-β near the tumor-vasculature bestowed slower-cycling properties to neighboring squamous cell carcinoma stem cells, which transcriptionally activates p21 and stabilized Nrf2 thereby markedly enhances glutathione metabolism to diminish the effectiveness of cisplatin^[125]^. In addition, TGF-β can induce EMT-mediated resistance to EGFR-TKI by activating the MAPK, which can be blocked by the MEK1/2 inhibitor^[126]^.

#### Drug resistance induced by overexpression of EMT-TFs

EMT-TFs such as Snail, Slug, Twist, Zeb, FOXM1 and FOXC2 act as bridges connecting EMT and drug resistance. One major mechanism is the overexpression of EMT-TFs enhances the efflux activity of ABC transporters and thereby promotes MDR. EMT-TFs are known to have binding sites on the promoter regions of multiple ABC transporter genes^[127]^. In sorafenib-resistant HCC with increased metastasis and enhanced MDR by upregulated P-gp, MDR was suggested as a downstream event from EMT and directly triggered by EMT, as the siRNA knockdown of Snail blocked EMT and partially reversed MDR^[128]^. PI3K/Akt signaling pathway can induce EMT-associated resistance by the direct regulation on Snail, which then activates P-gp-mediated MDR^[128,129]^. Overexpression of Slug in HCC cells induced chemo-resistance via upregulation of BCRP but downregulation of P-gp, as well as by the increased expression of stem cell marker CD133 in both complete and partial EMT phenotypes^[130]^. Chemotherapeutics like doxorubicin can induce FOXM1, which directly activates BCRP to increase the drug efflux and resistance in bladder cancer cells^[131]^.

Moreover, EMT-TFs can enhance antiapoptotic or suppress apoptotic signaling, either independent of or dependent on their role in the induction of CSC phenotype. For example, Twist1 induces resistance to EGFR-TKI by transcriptional suppression of pro-apoptotic gene *BCL2L11 *(BIM), and the addition of a pan-Bcl-2 inhibitor overcame the resistance^[132]^. Prostate cancer cells resistant to androgen deprivation (AD) therapy were characterized by elevated FOXC2, the associated EMT/CSC phenotype and increased drug resistance, which were related to the activation of p38 MAPK signaling, a key pathway in promoting cell survival and proliferation^[133]^.The study of the mechanism suggested that FOXC2 augmented p38 phosphorylation^[133]^.

Furthermore, EMT-TFs increase DNA damage repair and promote tumor cell survival under anticancer treatment. Overexpression of transcription factor 4 promoted doxorubicin resistance and stemness, likely by the upregulation of Zeb1 and Zeb2 in CRC cells^[134]^. Zeb1 promoted DNA repair via ATM- checkpoint kinase 1 (CHK1)- the mediated mechanism and was required for the clearance of DNA breaks, thereby inducing resistance to chemotherapy and radiotherapy^[135]^. Overexpression of Zeb1 activated ATM kinase by recruiting the transcriptional coactivators p300/PCAF to the ATM promoter, thus rendering chemoresistance in breast cancer cells^[136]^. In addition, the activated ATM was previously described to phosphorylate and stabilize Zeb1, indicating a positive feedback loop between Zeb1 and ATM regulation^[136,137]^. Activation of YAP and FOXM1 axis induced EMT-associated EGFR-TKI resistance in lung cancer by dysregulating mitosis. This was indicated by an increased abundance of spindle assembly checkpoint components including polo-like kinase 1, aurora kinases, survivin, and kinesin spindle protein^[80]^. FOXM1 was suggested as a potential biomarker for resistant lung cancers and its presence predicted a worse clinical outcome^[80]^.

### EMT is associated with activation of alternative pathways in acquired resistance to TKIs

Besides the resistance to conventional chemotherapeutics, EMT also contributes to the resistance of EGFR-targeted agents in many types of cancer cells^[138]^. Other than metabolic changes such as ROS or environmental cues like hypoxia, EMT can be induced by the activation of alternative pathways involved in acquired resistance to EGFR-TKIs, and the induced EMT further exaggerates the resistance. Poh *et al.*^[139]^ reported a case of EMT causatively inducing acquired resistance to the second-generation EGFR-TKI, afatinib in a patient with advanced non-small cell lung cancer (NSCLC), but negative for a secondary EGFR mutation. AXL is a receptor tyrosine kinase and its correlation with EMT has been demonstrated in NSCLC, breast cancer, and pancreatic cancer^[140]^. Acquisition of EMT phenotypes and AXL kinase activation were reported in the osimertinib-resistant cell line^[140]^. The insulin-like growth factor (IGF) signaling is crucial in growth, development and apoptosis. Upregulation of IGF1 receptor (IGF1R) was found to correlate with EMT and EGFR-TKIs resistance^[141]^. Notably, either silencing of IGF1R or direct inhibition of EMT by overexpression of E-cadherin substantially reduced EMT and resistance^[141]^. The mechanistic study suggested the activation of MAPK/ERK pathway downstream IGF1R induced EMT, which is indicated by increased nuclear β-catenin and Snail; the induction of EMT is associated with promoted resistance to EGFR-TKIs^[141]^. Sato *et al.*^[142]^ reported a feedback loop between MEK/ERK and PI3K/Akt pathways in EGFR-TKI-resistant NSCLC cell lines, suggesting resistance to EGFR inhibition may be a result and also a cause of the activation of PI3K/Akt-induced EMT. In advanced hepatocellular carcinoma, the sorafenib-activated Akt is thought to account for EMT and resistance to sorafenib via EMT-related upregulation of P-gp^[107]^. Treatment with a combined inhibition of MEK and PI3K pathways reversed EMT to MET and restored sensitivity to EGFR-TKIs^[128,142]^.

Inhibiting EMT to reduce drug resistance can be achieved by targeting characteristics associated with EMT, including TGF-β signaling, mesenchymal markers (vimentin, Slug, and Snail), AXL, EGFR^[143,144]^, and IGF signaling^[141,145]^. The development of therapeutics has been intensively reviewed^[143,144]^. In our recent study aiming at understanding the mechanistic roles of eATP in promoting EMT, we compared the gene expression changes induced by eATP and TGF-β, which would identify potential therapeutic targets (e.g., Stanniocalcin-1) involved in EMT and drug resistance. Table 2 is included below to summarize factors/pathways described in this section.

**Table 2:** Major factors and pathways involved in EMT-related drug resistance in cancer

**Involved factor**	**Normal biological function**	**Functions in cancer drug resistance**	**Major Ref.**	**Additional remarks**
**CSC induction**	Not applicable	Promoting stemness	[52,70,118]	
**TGF-β**	Inducing EMT and metastasis	Inducing drug resistant state of EMT and CSC	[120-126]	A master inducer of EMT and CSC
**EMT-associated ** **transcription factors**	Expression of EMT-related genes	Upregulating drug transporters, anti-apoptosis Enhancing DNA damage repair	[127-131] [132,133] [134-137]	Snail, Slug, Twist, Zeb, FOXM1 and FOXC2
**Alternative RTK signaling **	Cell growth, proliferation and survival	Activating EMT and related resistance	[128,140-142]	AXL, IGF, MEK/ERK and PI3K/Akt

## PATHWAYS CONNNECTING EMT AND CSC AND THE IMPLICATION IN DRUG RESISTANCE

Many recent studies suggest that EMT and CSC are intimately interconnected and their involvements in drug resistance represent different manifestations of a combined EMT/CSC phenotype^[11,70,146]^. One possible mechanism is that the activation of EMT and induction of CSC during tumor development are associated with activation of numerous signaling pathways that control CSC functions including self-renewal, differentiation, survival and metastatic potential. Among them, the Akt, Notch, Hh, Wnt/β-catenin, and NF-κB signaling pathways are responsible for inducing drug resistance. A better understanding of the mechanistic linkage between EMT, CSC and drug resistance would smooth the path for the identification of anticancer targets and promote therapeutic efficacy.

### Akt

Akt promotes cell survival, cell proliferation, induction of EMT and maintenance of stemness, it is considered as the master regulator of drug resistance^[147]^. Dysregulation of PI3K/Akt signaling prevails in human cancer and Akt often interacts with other signaling pathways involved in EMT/CSC to confer drug resistance. For instance, hypoxia synergistically enhanced gemcitabine-induced interaction between Akt and notch1, which in turn promoted cell stemness and chemoresistance^[148]^. Akt/glycogen synthase kinase 3-β activated Wnt signaling and promoted stemness and cisplatin resistance^[149]^. Akt cross talks with MAPK and NF-κB inducing overexpression of P-gp and MDR in cancer cells with EMT/CSC phenotypes^[128,150]^, as well as activating programmed death-ligand 1 expression which leads to immunosuppression^[147]^. Under hypoxia, PI3K/Akt coupled with mTOR and activated Hif-1α and Hif-2α, which promoted quiescence and chemoresistance via ABC transporters^[79]^. Combined therapy using PI3K inhibitor with inhibitors of other pathways that interact with AKT, like MAPK, might be a potent therapeutic strategy against drug resistance^[142]^.

### Notch

Notch signaling plays a crucial role in cell to cell communications, cell fate, angiogenesis, and CSCs of several tumors^[151,152]^. Notch signaling consists of two types of membrane-bound ligands: Delta-like ligands, and Jagged ligands. The binding of Notch ligands with four trans-membrane notch receptors (notch 1-4) leads to coordinated communication between adjacent cells^[151]^. Firstly, Notch cross talks with EMT: Notch induces EMT by upregulating EMT-TFs such as Twist, Snail, Slug, and Zeb; while Notch can be regulated by growth factors relevant to EMT such as fibroblast growth factor (FGF) and PDGF^[138]^. Further, Notch is an important mediator of CSC-related chemoresistance. For instance, CD133^+^/CD24^+ ^high renal cell carcinoma CSCs showed enhanced notch signaling, and blockage of Notch-1 or notch-2 reversed the stemness, invasiveness, migratory potential, and chemoresistance to cisplatin and sorafenib^[153]^. Also, Notch directly activates the transcription of the anti-apoptotic survivin, which serves as a Notch effector and contributes to CSC maintenance^[154]^. Moreover, activation of Notch signaling enhances the efflux activity of ABC transporters and promotes MDR. In NSCLC, Notch-1 contributes to chemoresistance via a Notch-1/AP-1/miR-451/MDR-1 axis, and inhibition of Notch-1 led to increased miR-451 and sensitized tumors to taxane-based treatment^[155]^. Patients of Stage II CRC with overexpression of hairy and enhancer of split-1 (HES1), a downstream transcriptional factor of Notch, showed higher recurrence rates after chemotherapy^[156]^. The over-expressed HES1 was found to correlate with increased MRP1, MRP2 and P-gp expression and chemoresistance in CRC cells^[156]^. Finally, Notch mediates resistance to EGFR-targeted agents by enhancing DNA damage repair and/or activating pro-survival signaling pathways. For instance, a combined blockade of EGFR and Notch-2/3 prevented acquired resistance to EGFR inhibitors and radiation, by reducing EGFR-TKI-induced ALDH^+^ and radiation-induced CD133^+^ stem cell subpopulations and expression of EMT and DNA repair genes^[157]^. In another example, Notch1 was necessary to induce trastuzumab resistance in breast cancer *in vitro* and *in vivo*^[158]^. Notch1 suppressed PTEN expression to activate ERK1/2 signaling, leading to breast CSC survival and bulk cell proliferation^[158]^. High Notch-1 protein expression was suggested as a marker to predict poorer survival in women with HER2^+^ breast cancer^[158]^. In gastric cancer cells, long-term treatment with trastuzumab resulted in upregulation of Notch ligand Jagged-1, leading to acquired resistance with EMT and CSC phenotypes through an IL-6/STAT3/Jagged-1/Notch positive feedback loop^[159]^. STAT3 is a well-known major cell survival factor and its activation in this axis contributed to the enhanced survival signaling and resistance to trastuzumab^[159]^.

### Hh

Hh signaling is central to embryogenesis, hyperactivation of this pathway has been found to be tumorigenic in many cancers^[151]^. Binding of Hh ligand to its receptor, Patched induces the activation of Smoothened (SMO) and Gli1; the SMO-mediated translocation of Gli1 into the cell nucleus drives the transcription of Hh target genes^[151]^. Hh signaling plays a key role in the maintenance of CSCs and chemo-resistance. In a study with an organoid culture model from patient-derived colorectal tumors, treatment with Hh inhibitors reduced expression of stem cell markers c-Myc, CD44, and Nanog, together with their transcription factor Gli1^[160]^. Combined treatment of Hh inhibitors with chemotherapeutics decreased the cell viability of organoids compared with the treatment of a single drug^[160]^. Finally, Hh signaling promotes drug resistance by inducing overexpression of P-gp and BCRP^[161]^. Approved SMO inhibitors are capable of reversing chemoresistance in gastric cancer and basal cell carcinomas^[161]^. Likewise in ovarian cancer cells, Hh signaling enhanced resistance to cisplatin, which was probably mediated by Hh transcription factor Gli2 which activated gene expression of P-gp^[162]^. Besides, BCRP was reported to mediate drug resistance in hepatoma cells under the transcriptional control of Gli1/2, and the inhibition of Gli1 or *ABCB2* gene expression ameliorated resistance to various chemotherapies in hepatoma cells^[163]^. Other ABC transporters were also found to confer chemoresistance as transcriptional targets of Hh/Gli signaling in CRC cells, including *ABCA2*, P-gp, *ABCB4*, *ABCB7*, *ABCC2* and *ABCG1*^[164]^. These findings suggest a connecting role of Hh signaling in CSC, EMT and chemo-resistance.

### Wnt

Wnt signaling helps determine cell fate during embryogenesis and regulates the homeostasis of many tissues in adults^[151]^. Aside from its function in normal cells, Wnt also plays a major role in maintaining CSCs in various cancer types. The binding of Wnt to frizzled receptors initiates two distinct signaling cascades, termed canonical or noncanonical. The activation of the canonical pathway results in translocation of β-catenin into the nucleus which transcriptionally controls a set of genes involved in cell migration [matrix metallopeptidases (MMPs)], survival (Myc) and proliferation (peroxisome proliferator-activated receptors-δ). Unlike the canonical pathway, the non-canonical pathway does not involve β-catenin^[151]^. Wnt signaling contributes to therapeutic resistance in various cancers by inducing several factors associated with CSC-like phenotypes. Radiation, chemotherapy and targeted therapy can stimulate Wnt signaling, which protects cancer cells from cell cycle arrest or apoptosis. Such apoptosis-protective effects are attributable to survivin and c-Myc, two downstream effectors of Wnt/β-catenin signaling^[165]^. Moreover, Wnt/β-catenin signaling is associated with drug resistance due to the overexpression of ABC transporters. Vesel *et al.*^[166] ^revisited cisplatin-induced multidrug resistance in NSCLC and found cisplatin up-regulated P-gp and BCRP by the induction of canonical β-catenin dependent Wnt7b signaling. Though P-gp and BCRP are not the drug transporters for cisplatin itself, they are transporters for drugs that are frequently used in combination with cisplatin (e.g., paclitaxel, doxorubicin and gemcitabine), putting forward the role of cisplatin in modulating drug response^[166]^. Next, Wnt signaling can prevent apoptosis by crosstalk with cell cycle checkpoint controls^[167]^. In chemoresistant CRC cells with a normal p53 pathway, the Wnt signaling inhibited CHK1 and suppressed CHK1-induced cell cycle arrest and apoptosis, leading to decreased stabilization of p53 and cell survival^[167]^. Furthermore, two recent studies reported that enhanced Wnt signaling promoted DNA damage repair and thereby induced resistance to the PARP inhibitor olaparib in ovarian cancers^[168,169]^. This mechanism may also protect cancer cells from DNA damage induced by other chemo- or radio-therapy^[170]^. Additionally, WNT/β-catenin serves as an alternative pathway in acquiring resistance to EGFR-TKIs. In preclinical studies, β-catenin was upregulated in CRC cells treated with BRAF inhibition by the activation of focal adhesion kinase^[171]^. Also in CRC cells, blocking Wnt/β-catenin signaling by tankyrase inhibitor enhanced the efficacy of the small molecular inhibitors targeting the PI3K and EGFR signaling pathways^[172]^. The TCF7 frameshift mutation in CRC cells induced aberrant regulation of WNT/β-catenin signaling and activation of GSK3, which promoted resistance to the dual PI3K/mTOR inhibitor gedatolisib^[173]^. Finally, Wnt signaling is one crucial player in the resistance to AD. Preclinical study results showed Wnt/β-catenin signaling was activated after AD and promoted androgen-independent growth^[174]^. Wnt activation induced expression of stemness markers (CD44, CD9, SOX10) which eventually reactivated androgen receptor (AR) signaling and contributed to enzalutamide resistance^[175]^. The strong correlation between AR and β-catenin levels can be used as a predictable indicator for resistance to chemotherapy during treatment^[175]^. Targeting the Notch, Hh, and Wnt pathways can control cancer stem cell replication, survival and differentiation as well as inhibit EMT, and thus reduce CSC-associated drug resistance^[176,177]^. Major approaches targeting Notch include γ-Secretase inhibitors and antibodies against the Notch receptor or ligand^[177]^. SMO inhibitors have been developed to target the signaling cascade of Hh^[177]^. Inhibition of the Wnt pathway can be achieved via neutralizing Wnt ligands or inhibiting the Wnt receptors, or targeting β-catenin by increasing its degradation or suppressing its function^[176,177]^.

### NF-κB

NF-κB is a well-documented mediator of the inflammatory response, during which the activation of NF-κB via toll-like receptors stimulation promotes stemness^[178]^, providing a link between inflammation and CSC phenotypes. Under hypoxic conditions, Hif-1α and Hif-2α induce the CSC features likely via NF-κB signaling pathway activation^[179,180]^. NF-κB is also suggested as a direct regulator of EMT-TFs and is associated with metastatic potential^[181]^. Resistance to radiotherapy correlates with increased EMT/CSC phenotypes induced via NF-κB^[182]^. Inhibition of NF-κB activity by metformin was associated with reduction of stemness and synergistically sensitized resistant lung cancer cells to EGFR-TKIs^[183]^. All the major pathways discussed in this section are summarized in Table 3.

**Table 3:** Major pathways connecting EMT with CSC in drug resistance in cancer

**Contributing factor**	**Normal biological function**	**Function in cancer drug resistance**	**Major Ref.**	**Additional remarks**
**Akt**	Cell growth and cell survival	Promoting stemness and related resistance	[79,128,147-149]	Crosstalk with other signaling pathways
**Notch**	Embryogenesis and tissue homeostasis	Inducing CSC and EMT, anti-apoptosis, promoting MDR	[138,153,157,159] [153] [155,156]	
**Hedgehog**	Embryogenesis and tissue homeostasis	CSC maintenance, promoting MDR	[160] [161-164]	
**Wnt**	Embryogenesis and tissue homeostasis	CSC maintenance, anti-apoptosis, enhancing DNA damage repair, promoting MDR	[176] [165,167] [168,169] [166]	Alternative pathway in resistance to EGFR inhibition
**NF-κB**	Gene expression, cytokine production Immune regulation	Promoting stemness, EMT and related resistance	[178-183]	Master inducer and regulator

### TME regulation on EMT, CSC and drug resistance

Drug resistance can be induced by non-cancer cells present within the TME. Environmental stress due to anticancer therapies can initiate cancer cell secretion of chemoattractants, which recruit various cells like macrophages into the TME and assist in the differentiation of the latter in a paracrine manner^[184]^. The newly differentiating non-cancer cells, such as cancer-associated fibroblasts (CAFs) and tumor-associated macrophages (TAMs), provide feedback to the cancer cells to promote stemness and resistance. CAF secretion of either exosome containing miRNA, Wnt proteins^[185]^, Interleukin (IL)-1B, TNFα or fibrillar collagen can activate EMT of cancer cells^[186]^. For example, exosome transfer of miR-92 from CAFs releases the inhibition of the Wnt/β-catenin pathway, which promotes stemness, EMT and chemoresistance^[187]^. CAFs can be activated by Hh ligand produced by tumor cells, leading to the expression of FGF5 and production of fibrillar collagen, which forms a supportive niche to facilitate cancer cells to acquire EMT/CSC phenotype and chemoresistance^[15]^. Combination of SMO inhibitor and chemotherapy improved response for 3 of 12 patients in a clinical trial^[15]^. TAMs can induce EMT and stemness of cancer cells and related treatment resistance by the secretion of Wnt proteins^[188]^, TGF-β, IL-6, IL-10 and TNFα^[189]^. In addition, The higher proportion of M2-like TAMS to M1-like TAMs creates an immunosuppressive TME possibly by releasing immunosuppressive cytokines, IL-10 and TGF-β, and activating induced regulatory T cells which inhibit the cytotoxic function of effector T and NK cells^[190,191]^. 

**Figure 1:**
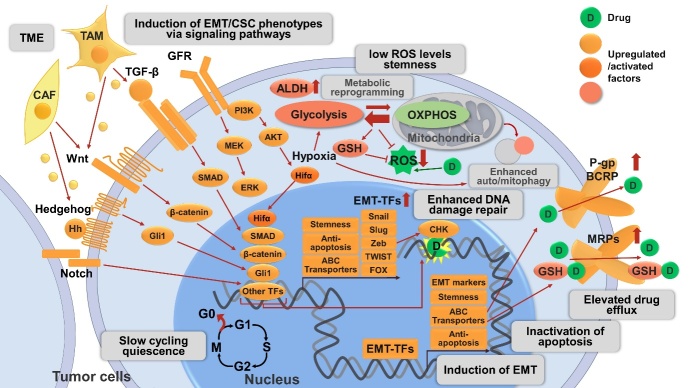
Mechanisms related to CSC and EMT involved in drug resistance in cancer described in this review. The CSC phenotype is a plastic state and can be adopted by most cancer cells. CSCs are slow-cycling or quiescent, and circumvent therapies targeting rapidly-dividing cells. CSCs exhibit increased activities of detoxifying proteins including ALDH and ABC transporters (P-gp, BCRP). CSCs maintain low levels of ROS by metabolic reprogramming and/or auto/mitophagy to protect from radio- and chemo-therapies. Hypoxia and Hifα are crucial regulators of metabolic reprogramming primarily by increasing flux to glycolysis and antioxidant production (e.g., GSH) and inducing autophagy. Hypoxia and Hifα are also emerging inducers of EMT/CSC phenotype, one of the mechanisms is via NF-κB signaling pathway activation (not shown in the figure). EMT is a crucial regulator of and tightly interconnected with CSC, their involvement in drug resistance may represent different manifestations of the EMT/CSC phenotype. Activation of diverse signaling pathways are involved in the induction of EMT/CSC phenotype, including developmental pathways (e.g., Wnt/β-catenin, Hh/Gli1, Notch), cell survival pathways (e.g., GFR), and EMT-related pathways (e.g., TGF-β/SMAD signaling). The above pathways act independently or cross talk with each other to induce EMT/CSC phenotypes, leading to elevated drug resistance by various mechanisms: (1) The activation of these pathways allows the maintenance of CSC properties, including enhanced drug resistance. (2) These pathways converge on EMT-TFs (e.g., Snail, Slug, Zeb, Twist, FOX, *etc*.) to alter the expression of EMT markers (e.g., increase in N-cadherin, vimentin; decrease in E-cadherin) and induce EMT; the downstream transcription factor of the above pathways, as well as EMT-TFs, can upregulate ABC transporters, leading to enhanced drug efflux. EMT-TFs also enhance stemness and anti-apoptotic signaling. (3) The activation of these pathways is associated with enhanced anti-apoptotic machinery and thereby promotes tumor cell survival. (4) Certain pathways and EMT-TFs like Zeb1 can enhance ATM and CHK1/2-mediated DNA-damage repair and promote resistance to genotoxic therapies. (5) Non-cancer cells such as CAFs and TAMs in the TME can also activate these signaling pathways by secreted proteins and thus promote drug resistance. CSC: Cancer stem cell; EMT: epithelial-mesenchymal transition; ALDH: aldehyde dehydrogenase; ABC transporters: ATP-binding cassette (ABC) transporters; P-gp: P-glycoprotein; BCRP: breast cancer resistance protein; ROS: reactive oxygen species; Hifα: hypoxia-inducible factors α; GSH: glutathione; NF-κB: nuclear factor-κB; Hh: Hedgehog; GFR: growth factor receptor; TGF-β: transforming growth factor-β; EMT-TFs: EMT-inducing transcriptional factors; Zeb: zinc finger E-box binding homeobox; FOX: forkhead box; ATM: ataxia telangiectasia mutated; CHK: checkpoint kinase; CAF: cancer-associated fibroblasts; TAM: tumor-associated macrophages; TME: tumor microenvironment.

All these discussed drug resistance mechanisms are schematically shown in Figure 1.

## ATP AND ATP-MEDIATED DRUG RESISTANCE

### Intracellular ATP promotes drug resistance

Adenosine 5’-triphosphate (ATP) is a multifunctional molecule participating in a myriad of cellular processes. ATP, as an energy supplier, signaling transducer, extracellular messenger, has been proven to be a significant player in tumor growth, survival and resistance^[192-195]^. Cancer cells are known to have higher levels of intracellular ATP (iATP) compared with their non-cancerous counterparts, likely due to the elevated glucose transport and aerobic glycolysis, namely the Warburg effect^[196,197]^. Notably, resistant cancer cell lines are found to have even higher iATP levels than their parental cancer cells, and the increased iATP levels have been demonstrated to contribute to the establishment and maintenance of resistance^[198]^. In colon cancer, direct delivery of ATP into cancer cells induced conversion of drug-sensitive cancer cells to drug-resistant cancer cells, while ATP depletion by glycolysis inhibition restored their sensitivity to chemotherapy^[198]^. This suggests iATP levels are a core determinant of chemoresistance^[198]^.

Accordingly, depleting iATP levels emerges as a promising strategy to restore drug sensitivity. Metformin, an AMPK activator and a prescription drug used to treat type 2 diabetes, significantly hampered the production of iATP, leading to a critical energy crisis which impaired the capability of the breast CSCs to repair chemotherapy-induced DNA damage *in vitro*. The addition of eATP completely abrogated such synergistic effects of metformin on the sensitivity to chemotherapeutics^[199]^. In one attempt, a covalent conjugate of nucleobases and peptides, or nucleopeptides, was engineered, which can selectively sequester ATP over ADP. This nucleopeptide slowed down efflux pumps in multidrug resistance cancer cells and boosted the efficacy of doxorubicin^[200]^. A recently described estrogen receptor α biomodulator, BHPI, is capable of inducing toxic hyperactivation of the endoplasmic reticulum stress sensor, the unfolded protein response. By this action, BHPI depleted iATP and nearly blocked P-gp-mediated drug efflux, which restored doxorubicin and paclitaxel sensitivity in ovarian cancer cells^[201]^. A novel nanoparticle was described, which independently encapsulated doxorubicin in the core and glucose oxidase (GOx) in the shell^[202]^. The fast release of GOx by acid-sensitive degradation of the shell consumed glucose and deprived ATP, which suppressed ATP-dependent drug efflux and facilitated the sequential accumulation of doxorubicin in breast cancer cells and dramatically improved the efficacy of anticancer drugs for MDR cells^[202]^.

### Extracellular ATP induces drug resistance

eATP levels of various cancer types are 10^3^ to 10^4^ times higher than those in their corresponding normal tissues^[1]^. The abnormally high concentration of eATP is majorly released from dying tumor cells, which can be induced by cellular stress, hypoxia, inflammation, platelet aggregation and anticancer therapies^[203]^. The functions of high eATP levels have been primarily revealed by our study. We investigated the role of eATP related to resistance: eight anticancer drugs, including both targeted and chemotherapeutic drugs, were tested in five cancer cell lines of five different organ origins. We found that eATP can promote the increase of iATP and cancer cell survival in most cases^[10]^. When studying promoted drug resistance to sunitinib due to eATP in NSCLC A549 cells, we saw that eATP can be internalized by cancer cells through clathrin- and caveolae-mediated endocytosis, but mainly by macropinocytosis indicated by colocalization of fluorescent-ATP with a macropinocytosis tracer (high molecular weight Dextran), resulting in substantially elevated iATP levels from 150% to 200% of the original iATP concentrations^[8-10]^. ATP levels increased by eATP is not a transient effect. The level elevation persists as long as the eATP is present^[9]^. Activation of macropinocytosis is a hallmark of some cancers, particularly for those harboring oncogenic Ras mutations^[204]^. We inhibited macropinocytosis by either siRNA knockdown of macropinocytosis-essential enzyme PAK1 or a macropinocytosis inhibitor IPA3, and the inhibition resulted in the reduction of iATP levels and survival of eATP and sunitinib treated A549 cells^[10]^. Thus, the elevated iATP level from macropinocytosis and other endocytosis-mediated eATP internalization is responsible, at least in part, for the observed drug resistance. One of the possible drug resistance mechanisms induced by the internalization of eATP is that more abundant iATP molecules compete with tyrosine kinase inhibitors, which are ATP competitors for the ATP binding site located on RTKs, leading to increased phosphorylation and activation of downstream signaling pathways^[10]^. Besides, the enhanced iATP is found to affect cancer cell metabolism, particularly those related to energy metabolism (unpublished observation).

In the five cell lines studied, increased iATP was found to correlate with drug resistance status when the ABC transporters expressed by the cell line matched those required for the efflux of a given drug^[10]^. Indeed, our results showed that the eATP molecules being internalized served as an energy supplement and enhanced efflux activity of ABC transporters in two cancer cell lines, resulting in increased cell survival^[10]^. In addition to directly providing energy, eATP was also shown to regulate expression levels of ABC transporters^[10]^. These results indicated the profound effects of eATP on modulating ABC transporter activity to potentiate efflux of anticancer drugs and enhancing drug resistance. Wang *et al*.^[205]^ reported a lipid membrane-coated silica carbon nanoparticle engineered to produce ROS in mitochondria under near-infrared laser irradiation. The introduced ROS species reduced iATP by oxidizing available NADH for ATP synthesis, and thereby suppressed the efflux of chemotherapeutics as well as reduced the expression of P-gp and its distribution on the plasma membrane, which is consistent with our findings^[205]^.

Additionally, our recent study demonstrated that eATP induced cancer cell migration and invasion. When exposing lung cancer cells to an ATP concentration equivalent to that in intratumoral extracellular space, we found accelerated detachment, EMT, migration, and invasion of lung cancer cells^[12]^. Mechanistically, we detected an increase in expression levels of MMPs, mesenchymal-phenotype molecules, EMT-TFs (vimentin, Snail and Slug) and decreased levels of epithelial phenotype markers (E-cadherin, β-catenin and ZO-1)^[12]^. Notably, these effects did not require TGF-β and were semi-independent of the activation of purinergic signaling (P2X7)^[12]^. Moreover, the knockout of a key macropinocytosis-associated gene, sorting nexin 5, significantly reduced micropinocytosis and the resulted iATP levels, cell growth, migration/invasion rates *in vitro*, and slowed down tumor formation and growth in nude mice^[12]^. As summarized in the previous content, EMT is intimately associated with the acquisition of resistance, and thus these results may reveal new mechanisms of the eATP-induced drug resistance. Overall, our studies suggested the multifunctional roles of ATP as an energy supplier facilitating drug efflux and a signaling transducer activating signaling pathways related to cell survival and resistance, drug efflux and tumor migration/invasion.

eATP can also contribute to cancer drug resistance as an extracellular messenger through P2 purinergic signaling, which is composed of nucleotides/nucleosides (mainly ATP and adenosine) acting as signaling ligands with their corresponding membrane receptors and modulating diverse signaling pathways^[206]^. There are two P2 receptor families: P2X receptors which are ATP-gated ion channels and P2Y receptors which are mainly activated by ATP or ADP. P2X and P2Y family members emerge as players in resistance to chemotherapies^[207,208]^. Ectonucleoside triphosphate diphosphohydrolase 1 (ENTPD1, or CD39) eATPase activity, which converts eATP to AMP, coordinately activated mitochondrial stress response via the downstream P2RY13/cAMP/PKA axis and promoted cytarabine resistance by enhancing mitochondrial OXPHOS activity in acute myeloid leukemia (AML). With such evidence, eATP was proposed as a key factor in chemoresistance of AML and CD39 as a new marker associated with a poor response to chemotherapy^[207]^. In the extracellular compartment, tumor cells can induce platelet activation and aggregation. Activated platelets release ADP and ATP, which activated ADP purinergic receptor P2Y12 expressed on pancreatic ductal adenocarcinoma and hence promoted expression of gemcitabine resistance markers Slug and cytidine deaminase (CDD)^[209]^. Exogenous ADP and ATP (100 µM) also increased the expression levels of Slug and CDD while P2Y12 inhibition completely blocked the survival signals initiated in cancer cells by platelet-derived ADP and ATP^[209]^. P2X7 receptor behaves as a bi-functional receptor. Tumor cell killing by mmol/L ATP concentration is in most cases mediated by the overstimulation of P2X7 and large pores opening on the plasma membrane. In contrast, low level of P2X7 stimulation often promotes cell survival and proliferation. P2X7 can potentiate OXPHOS, aerobic glycolysis and biosynthesis, which endows P2X7-expressing cells with a striking proliferative advantage^[210]^. A recent study described a distinct conformational form of P2X7, termed non-pore functional P2X7 (nfP2X7), which is unable to form a pore upon eATP stimulation. The exposure to a high ATP concentration (0.5-1 mM) drove nfP2X7 expression and was essential for tumor cell survival^[211]^. These results suggest a role of P2X7 in resistance to apoptosis-inducing chemotherapies. Besides, P2X7 expression is intimately associated with cancer cell metastatic potential and invasiveness^[210,212]^. In addition to P2X7, Some other purinergic receptors (PRs) are likely to be involved in drug resistance as well. These PRs have different EC50s compared with P2X7, they respond to different levels of extracellular ATP and mediates presently-poorly defined actions in drug resistance.

Nevertheless, several chemotherapeutic agents have the capacity to induce immunogenic cell death (ICD) which can lead to the generation of immunological memory and improve relapse-free survival^[213]^. In this scenario, the release of eATP from stressed or dying tumor cells during ICD is recognized as a damage-associated molecular pattern and is indispensable for the ICD^[213]^. For example, released eATP can ligate P2RX7 on dendritic cells to drive its recruitment and activation, which then generate adaptive anticancer immunity by the process of tumor-derived antigens and secretion of interleukin-1β^[214]^. eATP also executes immune-activating functions by communicating with other immune cells such as monocytes, macrophages, T cells, eosinophils, neutrophils and B cells^[203]^. Importantly, ICD depends not only on the release of eATP but also on its stability in the TME^[215]^. CD39 and cell surface purinergic enzyme ecto-5’-nucleotidase (NT5E, or CD73) can ultimately convert eATP to adenosine (ADO). While eATP promotes antitumor immunity, ADO attenuates immune response against tumors. Adenosine induced signaling would upregulate anti-inflammatory molecules, decrease anti-tumor function of immune cells (e.g., T cells, B cells, dendritic cells, mast cells, natural killer, macrophages) and activate immunoregulatory cells (e.g., regulatory T cells), shaping an immunosuppressive environment and diminishing the effect of immunotherapies^[203,216]^. In addition, autophagy of tumor cells is required for chemotherapy-induced ICD, in which it facilitates the release of eATP over the generation of adenosine^[217]^.

Therefore, inhibition of purinergic signaling might improve the response to anticancer drugs. Among purinergic receptors, P2X7 receptor is a potential candidate, as the application of its antagonists was suggested in the inhibition of tumor growth and migration^[203]^. To limit the conversion from eATP to adenosine, CD39 and CD73 emerge as targets for anticancer immunotherapy^[213]^. One study reported CD39 blockade augmented eATP/P2X7-mediated proinflammatory antitumor responses and the ultimate release of IL18, which facilitated the significant expansion and infiltration of intratumor effector T cells, and reversed anti-PD-1 resistance^[208]^. Furthermore, depletion of eATP may also reduce drug resistance. One novel approach described nanoparticles that can simultaneously release Fe3+ and doxorubicin at the TME upon near infrared laser irradiation, and Fe3+ can deplete eATP through metal ion-triphosphate coordination, sensitizing chemotherapy^[218]^. The potential inhibitory approaches may include pH-dependent activation of ATPase introduced in a prodrug form to reduce TME eATP levels or by inhibiting pathways or factors downstream from eATP’s purinergic receptor signaling. The former approach is based on the observation that the TME is more acidic compared to the normal tissues while the latter strategy relies on the identification and testing of key players in the eATP induced EMT and CSC. Finally, inhibition of macropinocytosis can reduce cell uptake of eATP and thus iATP levels, which might alleviate the drug resistance and EMT promoted by eATP.

**Figure 2:**
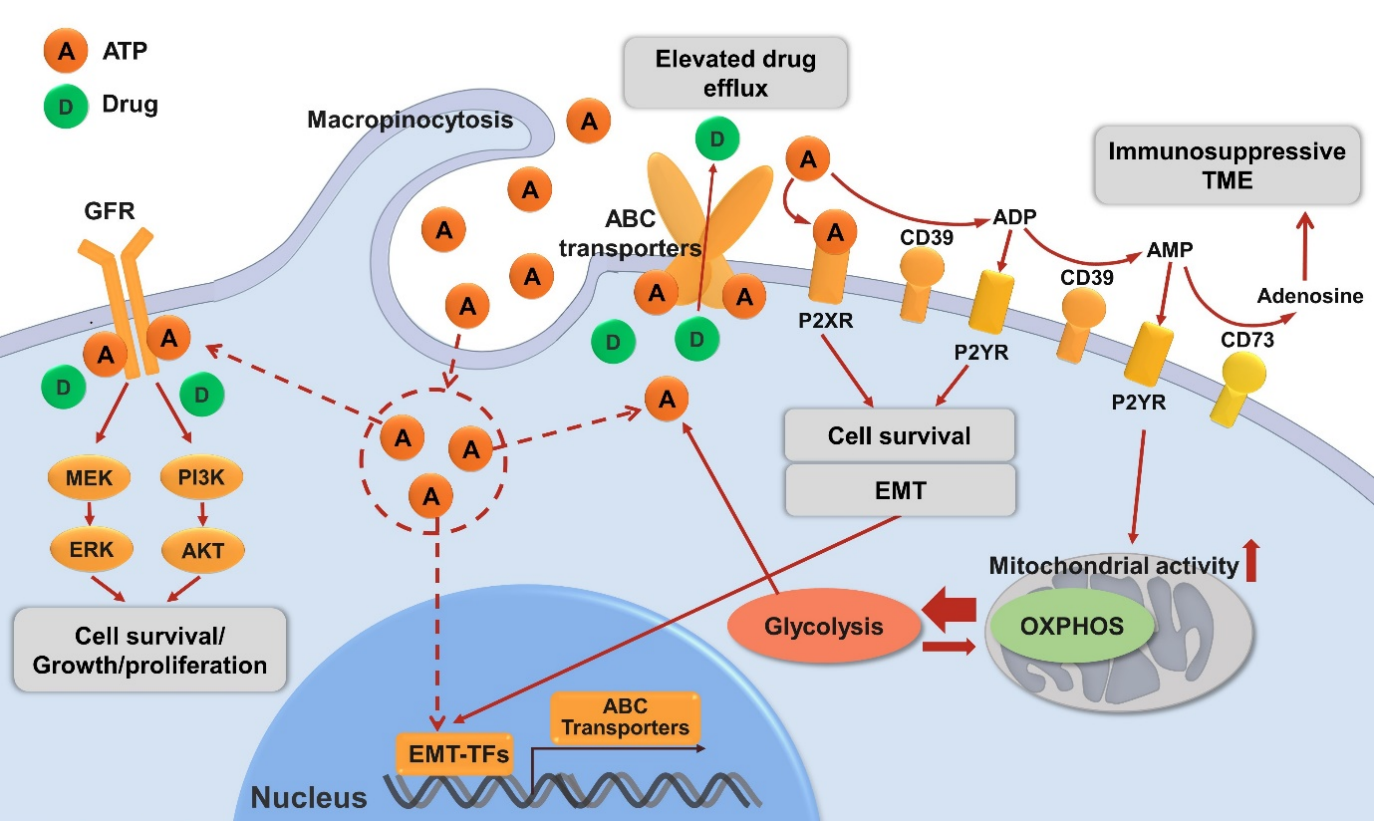
Mechanisms of drug resistance in cancer related to ATP described in this review. eATP can be degraded to ADP, AMP by ecto-nucleotidase CD39 or sequentially to immunosuppressive adenosine by ecto-nucleotidase CD73. eATP acts as messengers outside of cancer cells through purinergic signaling including P2X receptor (P2XR, e.g., P2X7) while ADP/AMP act through P2Y receptor (P2XR, e.g., P2Y12, P2Y13) to promote cell survival signals, energy generation or/and EMT, contributing to drug resistance. eATP is also internalized by cancer cells via macropinocytosis, which results in significantly elevated iATP levels. The increased iATP molecules become more competitive against ATP analog anticancer drugs for the intracellular ATP binding domain of RTKs of GFRs on cancer cell plasma membrane, and thereby reduce RTK phosphorylation and downstream signaling involved in cell growth, proliferation and survival. Elevated iATP levels also enhance the efflux activity of ABC transporters for out-pumping anticancer drugs from cancer cells. Additionally, our recent study identified a role of iATP directly inducing EMT, and EMT confers drug resistance by numbers of mechanisms including upregulating ABC transporters. All these mechanisms work together to promote drug resistance by increasing cell survival signaling, reducing intracellular drug concentration, and inducing EMT. Further studies are needed for the final validation of ATP-mediated mechanisms of drug resistance. eATP: Intratumoral extracellular ATP; ENTPD1: ectonucleoside triphosphate diphosphohydrolase 1 (CD39); NT5E: ecto-5’-nucleotidase (CD73); EMT: epithelial-mesenchymal transition; iATP: intracellular ATP; RTK: receptor tyrosine kinase; GFR: growth factor receptor; ABC transporters: ATP-binding cassette (ABC) transporters.

All these discussed ATP-related drug resistance mechanisms are shown in Figure 2.

## CONCLUSIONS

Through this review, we show that eATP, which has been found to be present in TME with concentrations in hundreds of micromolar, is an emerging inducer and regulator of drug resistance in cancer. eATP has been shown by us and others to be an inducer and regulator for EMT, which is closely associated with the formation of increasing CSC subpopulations in tumors. EMT/CSC phenotypes work together to increase therapy resistance by various mechanisms discussed above. Thus, eATP in TME is a factor, along with TGF-β, working through inducing EMT and CSC, and leading to a drug resistance state in cells and tumors. To reduce drug resistance and enhance anticancer therapeutic effects, inhibiting and blocking eATP is likely to be a promising approach^[219]^.

## DECLARATIONS

### Copyright

© The Author(s) 2021.
